# An intra-bone axial load transducer: development and validation in an in-vitro radius model

**DOI:** 10.1186/s40634-015-0035-z

**Published:** 2015-11-04

**Authors:** Nikolas K. Knowles, Michael Gladwell, Louis M. Ferreira

**Affiliations:** Roth|Mcfarlane Hand and Upper Limb Centre, Surgical Mechatronics Laboratory, St. Josephs Health Care, 268 Grosvenor St., London, N6A 4V2 ON Canada; Schulich School of Medicine and Dentistry, The University of Western University, 1151 Richmond St., N6A3K7 London, ON Canada; Department of Biomedical Engineering, The University of Western Ontario, 1151 Richmond St., N6A3K7 London, ON Canada

**Keywords:** Axial load transducer, Radiocapitellar joint, Bone-bridge, Radial head, Forearm loading, Radioulnar load sharing, Joint loading

## Abstract

**Background:**

Accurate measurement of forces through the proximal radius can assess the effects of some surgical procedures on radioulnar load sharing, but is difficult to achieve given the redundant loading nature of the musculoskeletal system. Previously reported devices have relied on indirect measurements that may alter articular joint location and function. An axial load transducer interposed in the diaphysis of the radius may accurately quantify unknown axial loads of the proximal radius, and maintain articular location.

**Methods:**

An *in-vitro* radius model was developed by interposing an axial load transducer in the diaphysis of the proximal radius. Static loads of 20, 40, 60, 80, and 100 N were applied with a servo-hydraulic actuator to the native radial head at angles of 10°, 20°, 30°, and 40° in the anterior, posterior, medial and lateral directions.

**Findings:**

Linear regression of five repeatability trials showed excellent agreement between the transducer and applied loads (*R*^2^ = 1 for all trials). For off-axis net joint loads, the majority of measured loading errors were within the inter-quartile range for mean loads up to 80 N. Loads below 80 N and outside the inter-quartile range had errors of less than 1 N.

**Conclusions:**

The repeatability and off-axis net joint load results of this study validate the effectiveness of the interposed axial load transducer to accurately quantify proximal radius loads. The surgical technique preserves the native articular location and soft-tissue constructs, like the annular ligament. The modular design allows for testing the effects of length-changing osteotomies in subsequent biomechanical studies.

## Introduction

Load-sharing between the radius and ulna can be altered by some orthopaedic procedures as a result of implant placement during total elbow arthroplasty, replacement of the radial head with a prosthesis, or radial head and capitellum surgery (Markolf et al. 1998). In many clinical cases, length changes of the radius occur (Markolf et al. 1998), altering loads through the articulation and soft tissues. Length changes of the radius alter the natural kinematics of the elbow, intuitively increasing or decreasing radioulnar load sharing as a function of increasing or decreasing radial length. Although it has been suggested that failure to restore the joint to its native condition has unknown consequences (Shaaban et al. 2006), altered loads may increase the risk of cartilage wear and degenerative changes at the radiocapitellar and ulnohumeral articulations. As such, post-traumatic arthritis has been well-described and is a common complication of complex elbow injuries, even following appropriate bone and ligament repair (Ring et al. 2002). To ensure native load-sharing is maintained following surgical procedures to minimize the risk of the aforementioned suboptimal outcomes, it is essential to isolate intra-articular loads. Isolating loads through the proximal radius is impossible without interposing a measurement device within the bone due to the redundant loading scenario imposed by soft-tissues and osseous contacts.

Multiple studies have assessed elbow load sharing, and describe devices capable of measuring in-vitro radial and ulnar loads (Ekenstam et al. 1984; Markolf et al. 1998; Palmer et al. 1984; Palmer and Werner 1981; Pfaeffle and Fischer 1999; Rabinowitz and Light 1994; Trumble and Glisson 1987); however these devices disrupt soft tissues, especially the annular ligament, which are significant contributors to the net load vector through the radial head. Such a device which does not disrupt soft tissues, or alter the native location of the radial head, has yet to be described. This study describes the validation of a custom axial load transducer implanted in the proximal radius. The device was tested in an in-vitro radius model with a servo-hydraulic testing frame to assess the devices accuracy in measuring a wide range of proximal radius loading vector magnitudes and directions. Following validation of the device, its use in biomechanical studies assessing radiocapitellar and ulnar load sharing as a function of length changes of the radius will be performed.

## Methods

**Fig. 1:**
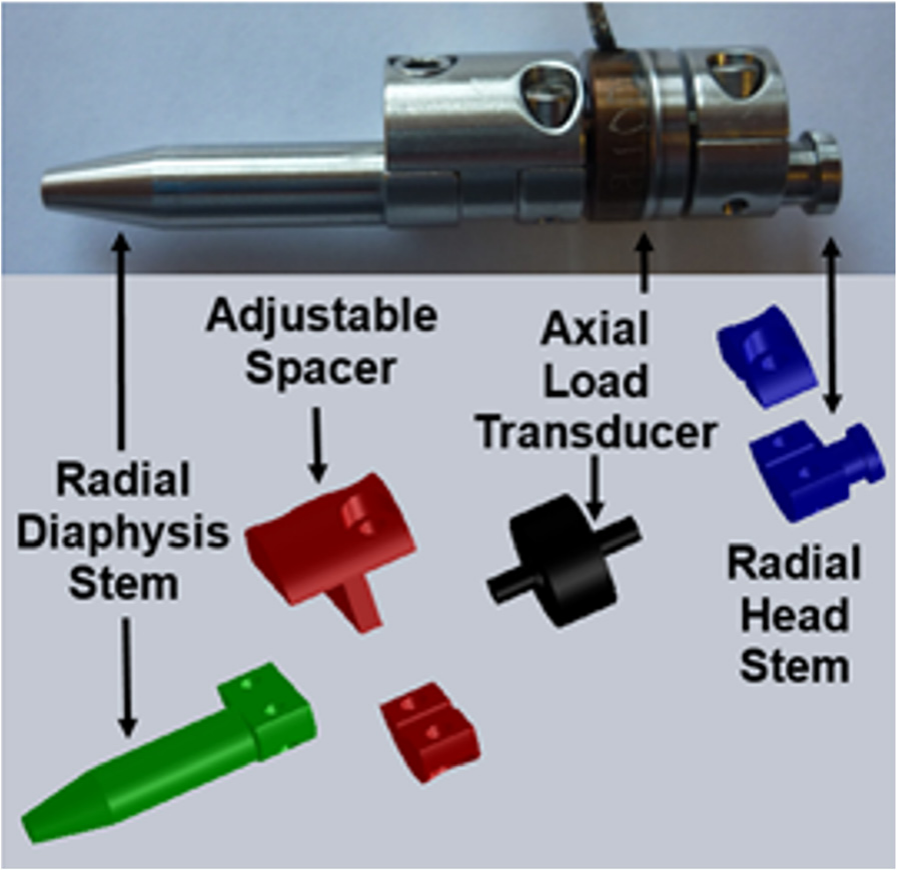
Modular axial load transducer components. The radial and diaphysis stems are cemented in-situ*.* The adjustable spacer was changed to assess the effect of lengthening and shortening in subsequent cadaveric biomechanical studies

The *in-vitro* model consisted of one fresh-frozen cadaveric radius (age: 62, male) and an interposed modular axial load transducer device (Fig. 1). The device was custom manufactured to allow for stem fixation in the diaphysis and head of the radius. An adjustable spacer was included as part of the design to allow for the effect of radial length changes on radial loads to be measured in subsequent cadaveric biomechanical studies. The device uses a commercially available axial load transducer (Subminiature Model 11, Honeywell, Morristown, NJ, USA) and the modular design allows for installation while maintaining a bone bridge to preserve the native radial head articular location.

**Fig. 2:**
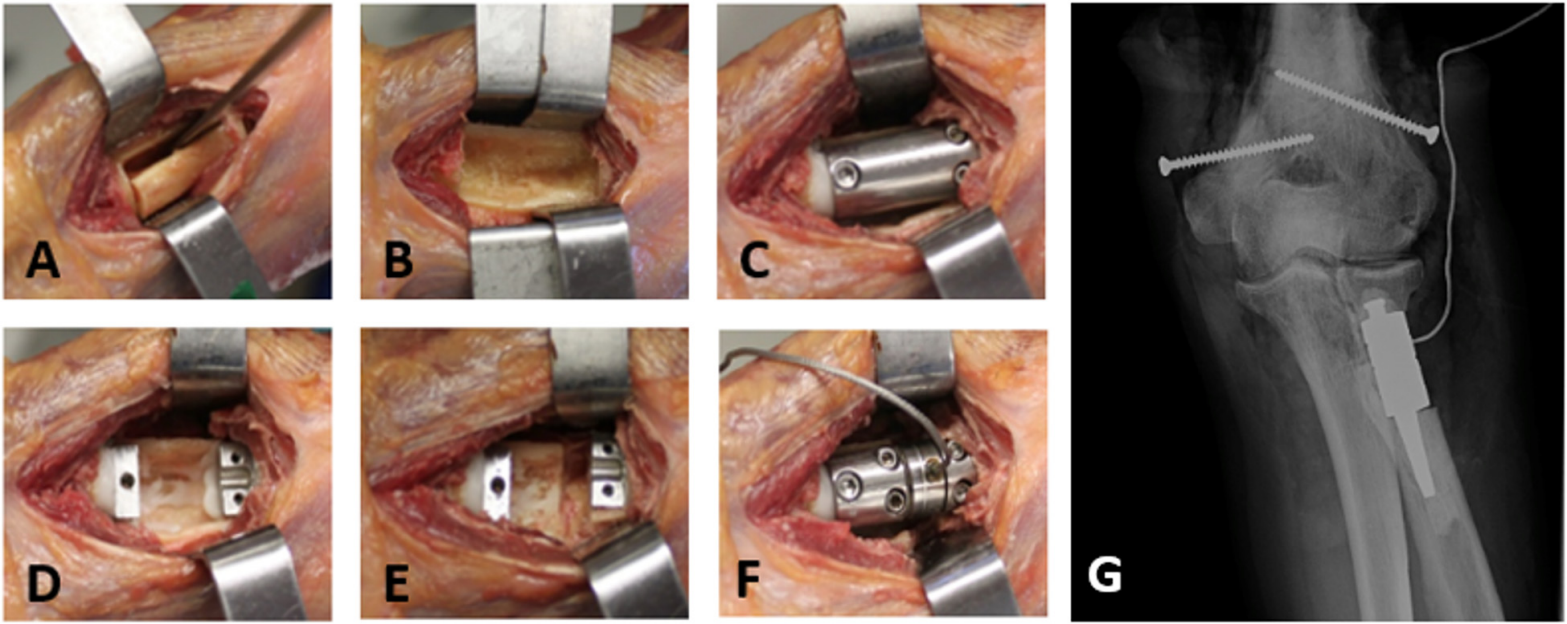
The bone-bridge surgical technique. Removal of cortical window (**a**). Exposed intermedullary canal (**b**). Head and diaphysis stem placement using spacer to ensure alignment (**c**). Spacer removed and stems cemented in-situ (**d**). Removal of bone-bridge (**e**). Implantation of load transducer and re-alignment of native anatomical position (**f**). Radiograph of implanted load transducer device in a cadaveric radius (**g**)

The surgical procedure (Fig. 2) consisted of first opening a cortical window in the radial neck to expose the intermedullary canal. Bone cement was then inserted in the diaphysis and radial head, the respective stems were placed, and the load transducer was assembled in-situ*,* prior to cement hardening. At this time a section of the radial neck remained, termed the ‘bone-bridge,’ in order to preserve the native radial head articular location. The proximal radius osteotomy was completed after the cement hardening by removing the bone-bridge. The radius was then excised from the forearm and denuded of all soft-tissue for validation testing of the implanted device.

The radius was rigidly mounted in a clamp for digitization of the load transducer and bony landmarks using a rigid body stylus with an optical tracking system (Optotrak Certus, NDI, Waterloo, CAN). These landmarks were used to derive an anatomical coordinate system about the long-axis of the radius. The anatomical coordinate system was derived using the mid-point of the dorsal and volar aspects of the distal radial ulnar joint (DRUJ), and radial styloid point, as well as the center of the radial head. The proximal and distal coordinates of the load transducer within the bone were also digitized to determine its relative offset with the anatomical long-axis. This coordinate system allowed for alignment of the anatomical long-axis relative to the actuator of a servo-hydraulic testing frame (Instron^®^ 8501, Norwood, MA, USA). Transducer offset was 3.61° in the anterior-posterior and 3.50° in the medial-lateral planes relative to the anatomical long-axis.

**Fig. 3:**
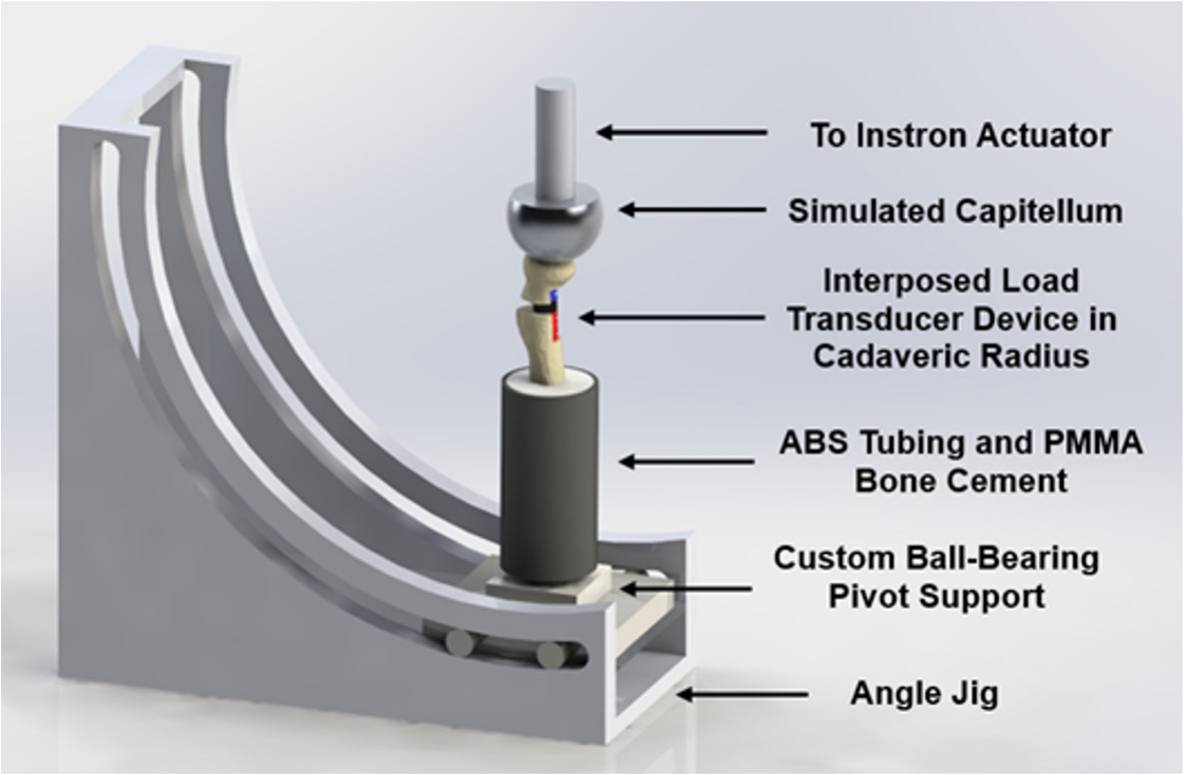
Experimental set-up of the in-vitro radius model with interposed axial load transducer. The angle jig was mounted to an X-Y stage on a servo-hydraulic testing frame (not shown) for alignment of the actuator and simulated capitellum with the radial head. Note the support base could be fixed at the desired angle along the angle jig

The radius was aligned and fixed in an ABS tube using the same previously digitized anatomical landmarks. Bone cement was placed in the ABS tube to secure the radius and maintain alignment of the anatomical long-axis. The ABS tube with the proximal radius protruding was mounted on a custom base within an angle jig mounted to an X-Y stage (Fig. 3). This jig allowed for a range of simulated net load vector angles of the model, and the X-Y stage ensured proper alignment of the servo-hydraulic actuator with the anatomical long-axis. The native capitellum diameter was simulated using a metal hemisphere mounted to the servo-hydraulic actuator. The base of the ABS tube pivoted on a smooth spherical bearing, aligned with the digitized anatomical long-axis of the radius, to nullify reaction moments.

**Fig. 4:**
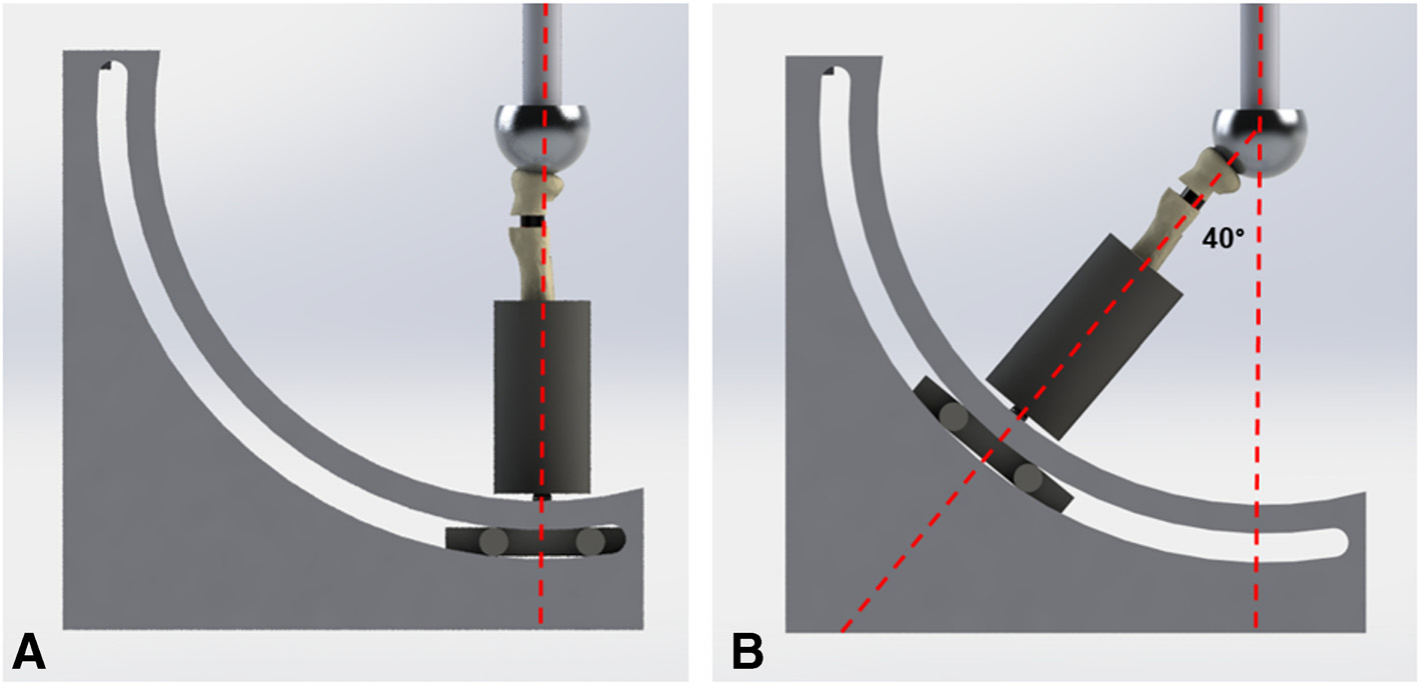
Alignment of the servo-hydraulic actuator with the anatomical long-axis of the radius. Zero degree applied load direction (**a**). The anatomical long-axis alignment of 40° to the servo-hydraulic actuator (**b**). Note that the radius was tested at angles of 10º, 20º, 30º and 40° and four rotations of the radius for net loads in the anterior, posterior, medial, and lateral directions

Repeatability was assessed using five independent trials with the anatomical long-axis aligned with the servo-hydraulic actuator (0° position) (Fig. 4a). Loads were applied at 10 Hz from 0 to 450 N and corresponding transducer loads were collected at 10 Hz. Linear regression was used to assess linearity of the applied load and measured transducer loads for each trial. This repeatability test represented a 4.5 fold increase in desired maximum validation testing load, and was also used to assess the durability of the device under extreme loading conditions.

For validation testing of a variety of simulated net joint load directions and magnitudes, static loads were applied from 20 to 100 N at 20 N increments at angles of 10°, 20°, 30° and 40° to the anatomical long-axis. The radius model was rotated in quarter turn increments to assess loads in the anterior-posterior and medial-lateral planes, resulting in 16 separate loading conditions. Axial load transducer values were collected using custom software (Labview, National Instruments, Austin TX, USA).

External loads of 20, 40, 60, 80, and 100 N were applied using the Instron servo-hydraulic actuator. The Instron load cell provided the ‘gold standard’ load measurement, which was corrected for the angle between the actuator applied load and the anatomical long-axis, to calculate the radial axis load (Fig. 4b). The transducer axis offset of 3.61° was added to the off-axis net joint load angle in the anterior direction and subtracted in the posterior direction, and transducer axis offset of 3.50° was added in the medial direction and subtracted in the lateral direction (i.e. for 10° off-axis load in the anterior direction, $\mathrm{radial}\ \mathrm{axis}\ \mathrm{load} = \frac{\mathrm{applied}\ \mathrm{load}\ \left(20,40,60,80,100\mathrm{N}\right)}{ \cos \left(10{}^{\circ} + 3.61{}^{\circ}\right)}$). The measured transducer loads and known radial axis loads were plotted as Bland-Altman plots to assess variations in transducer and Instron loads, and to visualize systematic bias. Correlations between load transducer and radial axis loads at each net load position were calculated using Pearson Product Moment Correlations. Significance was set at *p* < 0.05.

## Findings

Linear regression of the five repeatability trials with the actuator and anatomical long-axis aligned (0° position), showed linear trends for all five trails and both the measured load transducer and radial axis loads (*R*^2^ = 1 for all trials). There was no variation in the measured load transducer values with the 4.5 fold increase in maximum load, indicating the device’s ability to withstand forces greater than experienced in-vivo*,* without disruption to device fixation or the load transducer.

**Fig. 5:**
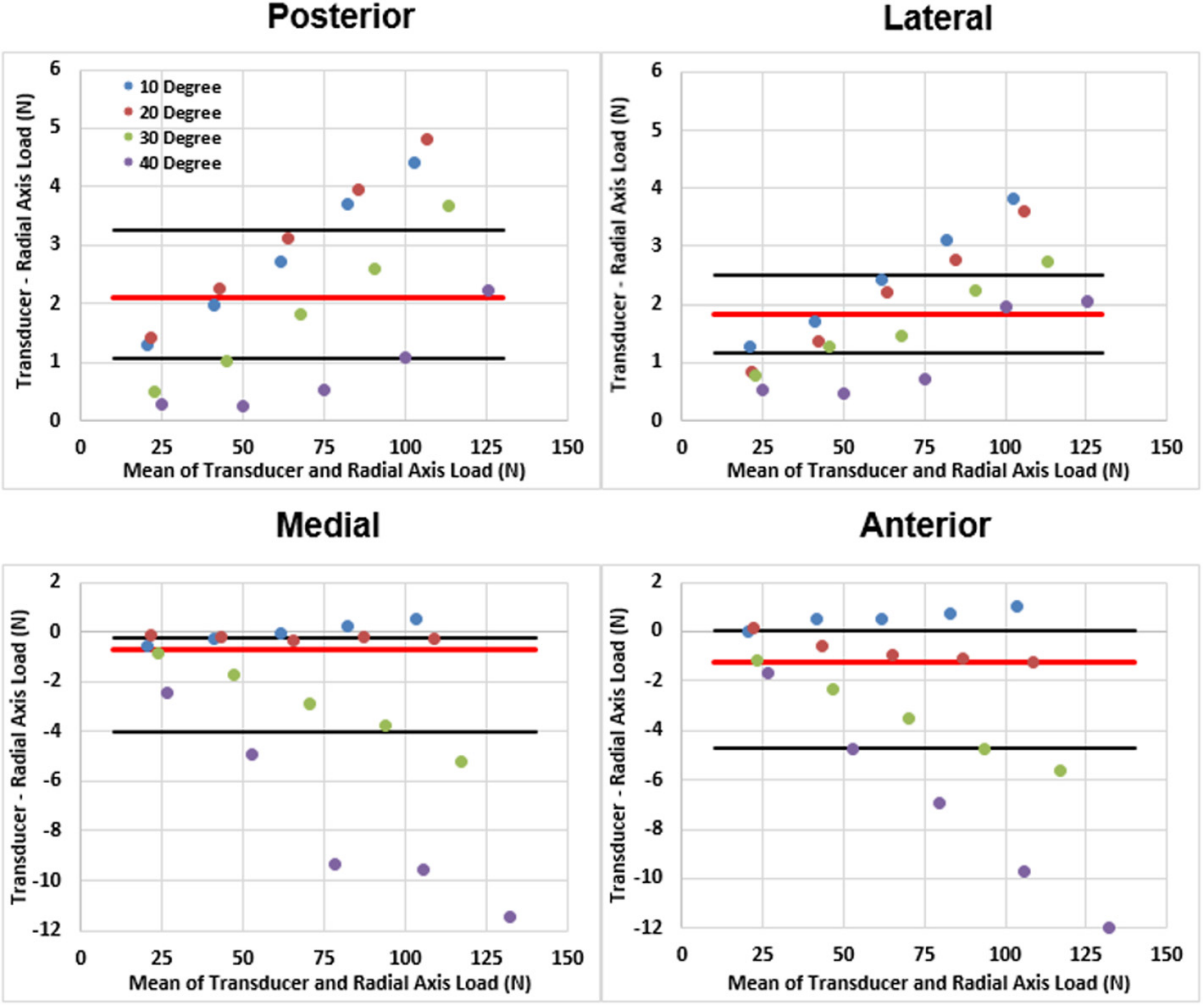
Bland-Altman plots of four load angles and four rotations of the in-vitro radius model. The red line indicates the median and the black lines indicate the 25th and 75th quartiles for the non-parametric data

Differences in transducer and radial axis loads were less than 6 N for off-axis angles up to 30° in all four load directions. Differences increased linearly for linearly applied static loads, and were greatest at applied loads greater than 80 N. Most differences in loads were within the inter-quartile range for mean loads of 80 N. Loads below 80 N and outside the inter-quartile range had load differences of less than 1 N. Pearson Product Moment Correlations showed significant relationships between transducer and radial axis loads for all 16 net joint load directions (*r* = 1.00 and *p* < 0.001 for each net joint load). Largest differences between transducer and radial axis loads occurred at 40° in the medial and anterior directions (Fig. 5).

## Conclusions

The majority of differences between transducer and known radial axis loads below load magnitudes of 80 N fell within the inter-quartile range for each net load direction. Loads below 80 N outside of this range had load magnitudes of less than 1 N. This result, as well as the results of the repeatability trials, validate the effectiveness of the interposed transducer device to accurately quantify intra-articular loading of the proximal radius.

It is important to keep in mind that the range of angles tested does not represent the joint range, but rather a range of loading vectors due to articular contact and soft tissue supports. This does not suggest that possible load vectors can be this extreme. As such, this validation shows that this device can accurately represent a wide cone range of internally generated net loads.

The medial and anterior loading directions are perpendicular, and as such the increased error in these positions cannot be explained by reduced implant fixation or stiffness of the overall construct. In accounting for misalignment of the load transducer measurement axis with the anatomical long-axis, it was noted that the transducer was positively misaligned in these directions. Although this misalignment was accounted for, and was constant throughout testing, it may interact with the load transducers off-axis measurement error to produce a non-linear response, contributing to the larger differences observed at these two positions.

Strengths of this study include the modular design of the axial load transducer, which allows for in-vitro real-time dynamic testing of radial length changes. The bone-bridge surgical technique ensures proper alignment of the device with the anatomical long-axis of the radius while maintaining the articular locations and soft-tissue constructs, such as the annular ligament. Furthermore, validation with the Instron servo-hydraulic testing frame and an external ‘gold standard’ load cell, provided a highly repeatable and controlled testing platform to measure loading of the interposed device. The ball-bearing pivot support nullified reaction moments and ensured only desired loads were measured.

Limitations of this study include the requirement to calculate the radial axis load partly based on the off-axis loading angle. Of course, it was not possible to directly measure the applied radial axis load because that was the novel function of the transducer. However, although the misalignment of the load transducer was determined with a highly accurate digitizer, any error in these angle measurements may have contributed a systematic bias.

This study provides a validated in-vitro interposed axial load transducer to be used in cadaveric biomechanical studies. Its modular design allows assessment of length-changing osteotomies, and its compactness provides for minimal soft-tissue disruption, including the annular ligament. Additionally, it allows preservation of the native articular alignment. Maintaining the native articulations using the bone bridge technique ensures physiologic loads are measured.

## References

[CR1] af Ekenstam F, Palmer AK, Glisson RR (1984). The load on the radius and ulna in different positions of the wrist and forearm: a cadaver study. Acta Orthop Scand.

[CR2] Markolf KL, Lamey D, Yang S, Meals ROY, Hotchkiss R (1998). Radioulnar load-sharing in the forearm. J Bone Joint Surg Am.

[CR3] Palmer A, Werner F (1981). The triangular fibrocartilage complex of the wrist—anatomy and function. J Hand Surg Am.

[CR4] Palmer AK, Werner FW, Eng MM (1984). Biomechanics of the distal radioulnar joint. Clin Orthop Relat Res.

[CR5] Pfaeffle HJ, Fischer K (1999). A new methodology to measure load transfer through the forearm using multiple universal force sensors. J Biomech.

[CR6] Rabinowitz R, Light T (1994). The role of the interosseous membrane and triangular fibrocartilage complex in forearm stability. J Hand Surg Am.

[CR7] Ring D, Jupiter J, Zilberfarb J (2002). Posterior dislocation of the elbow with fractures of the radial head and coronoid. J Bone Joint Surg Am.

[CR8] Shaaban H, Giakas G, Bolton M, Williams R, Wicks P, Scheker LR, Lees VC (2006). The load-bearing characteristics of the forearm: pattern of axial and bending force transmitted through ulna and radius. J Hand Surg Br.

[CR9] Trumble T, Glisson R (1987). Forearm force transmission after surgical treatment of distal radioulnar joint disorders. J Hand Surg Am.

